# Luspatercept‐induced reduction in transfusion requirement in α‐thalassemia

**DOI:** 10.1002/jha2.54

**Published:** 2020-07-09

**Authors:** Nicholas Jackson, Shasha Khairullah, Ping Chong Bee

**Affiliations:** ^1^ Clinical Haematology Unit Universiti Malaya Medical Centre Kuala Lumpur Malaysia

**Keywords:** erythropoiesis, TGF beta, thalassemia, therapy, transfusion

## 1 INTRODUCTION

Luspatercept is a fusion molecule combining a transforming growth factor‐β (TGF‐β) ligand scavenger with an immunoglobulin G1 Fc receptor. By ‘trapping’ excess TGF‐β ligands, such as GDF11, it promotes late erythroblast maturation, reducing ineffective erythropoiesis [1] which is the major cause of anaemia and many other problems in β‐thalassemia [2]. Capellini et al have recently reported the results of the BELIEVE trial, a randomised placebo‐controlled study, which showed that luspatercept reduces transfusion requirements in some patients with transfusion‐dependent β‐thalassemia [3]. However, the effectiveness of luspatercept has not yet been investigated in α‐thalassemia. The α‐thalassemias are generally mild, but the non‐deletional haemoglobin H (HbH) diseases, for example HbH with constant spring (HbH + CS), cause transfusion dependence in some patients [4]. Both deletional and non‐deletional HbH diseases are common in East Asia [5]. We have a patient with HbH + CS who was inadvertently entered into the BELIEVE study, and report her clinical response during 6 months’ exposure to luspatercept.

## 2 CASE REPORT

**FIGURE 1:**
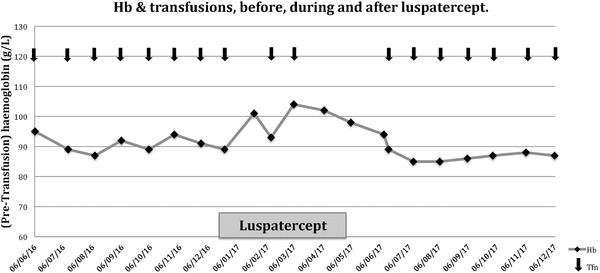
Hemoglobin values from 6 months before, to 6 months after, treatment with subcutaneous luspatercept 1.0 mg/kg every 3 weeks. One unit red cell transfusions are indicated by the arrows and were given every 4 weeks before and after treatment. During the 6 months of luspatercept treatment, the transfusions became less frequent and ceased for the final 3 months.

A Chinese Malaysian lady, currently 34 years old, had been diagnosed (erroneously, as it turned out) as β‐thalassemia major and transfused monthly from the age of 1 year. She underwent splenectomy at 18 years of age and developed hypogonadism, diabetes mellitus and osteoporosis. Her pre‐transfusion hemoglobin (Hb) averaged 91g/L, and she received one unit of blood monthly. In good faith she was entered into the BELIEVE study and started on investigational product. Later unblinding of the trial revealed that she did receive active agent (luspatercept 1mg/kg subcutaneously every 3 weeks). There was immediate improvement in her Hb, such that the next transfusion was delayed, and overall she received only two units over the next 6 months, with the second 3 months being transfusion free (Figure1). She felt marked symptomatic improvement (more energy and less breathlessness) and experienced no significant side effects. Her average Hb, taken monthly, was 99 g/L during the treatment. There were minimal increases in serum bilirubin (11 to 16μmol/L) and lactate dehydrogenase (139 to 158 U/L) during luspatercept treatment, suggesting a mild resurgence of HbH‐induced haemolysis. Her correct molecular diagnosis became known 6 months later, when the trial's centralised molecular analysis became available (HbH + CS, α‐genotype: ‐ ‐^SEA^/α^CS^α; with a normal β‐globin genotype). She was withdrawn from the study. In view of her response, a request was made for her to continue on luspatercept on a named‐patient basis. However, because of regulatory requirements, and a lack of safety data in α‐thalassemia patients, this request was turned down. Her Hb subsequently declined, her symptoms returned and she required monthly transfusions again. Her average pretransfusion Hb in the 6 months after cessation of luspatercept was 87g/L.

## 3 DISCUSSION

HbH (a tetramer of β‐globin) is a very high affinity Hb, which is of no value in delivering oxygen to tissues. In a typical patient with HbH disease, 5‐20% of the Hb is this useless Hb [6], meaning the patient may be symptomatic even with mild anaemia. This patient has a severe type of HbH disease, due to coinheritance of α^0^‐thalassemia trait and α^CS^ trait; such cases are often transfusion dependent from a young age, as in her case. We do not have access to her presenting clinical notes or results to explain why she was mistakenly diagnosed as β‐thalassemia major. Transfusion suppresses production of the HbH, and hence a higher proportion of the total Hb is functional. Interestingly, our patient had marked symptomatic improvement when she came off transfusion while receiving luspatercept, even though some of the increase in her Hb would have been HbH.

Members of the TGF‐β superfamily of ligands, including growth differentiation factors and activins, inhibit late‐stage erythropoiesis [7]. Luspatercept, a recombinant fusion protein consisting of a modified form of the extracellular domain of the human activin receptor type IIB linked to the human IgG1 Fc domain, scavenges these ligands [7, 8]. It thereby promotes more effective erythropoiesis, for example, in myelodysplasia [9] and has shown impressive activity in β‐thalassemia in Phase II [10] and III studies [3]. Luspatercept has not been formally tested in α‐thalassemia, as the predominant mechanism underlying anemia in this condition is extravascular hemolysis [4]. However, increased ineffective erythropoiesis may also play a role, especially in HbH + CS in which the abnormal αCS chains may be directly toxic to the red cell membrane leading to intramedullary apoptosis [11]. Our experience in this patient suggests that further studies of luspatercept are warranted in the severe α‐thalassemias, such as nondeletional HbH disease.

## CONFLICT OF INTEREST

The authors declare no conflict of interest.

## Data Availability

The data that supports the findings of this study are available from the corresponding author upon reasonable request.
